# New developments in GLP-1 agonist therapy for gestational diabetes: Systematic review on liraglutide, semaglutide, and exenatide from ClinicalTrials.gov

**DOI:** 10.1097/MD.0000000000044917

**Published:** 2025-10-03

**Authors:** Fahad S. Alshehri

**Affiliations:** a Pharmacology and Toxicology Department, College of Pharmacy, Umm Al-Qura University, Makkah, Saudi Arabia.

**Keywords:** gestational diabetes mellitus, GLP-1 receptor agonists, liraglutide, pregnancy pharmacotherapy, semaglutide

## Abstract

**Background:**

Gestational diabetes mellitus (GDM) is a widespread pregnancy complication, affecting approximately 7% to 10% of pregnancies worldwide and presenting risks for both maternal and fetal health. Traditional treatments, including lifestyle changes, insulin, and oral hypoglycemic agents, have limitations, particularly in terms of safety and potential fetal impacts. Glucagon-like peptide-1 (GLP-1) receptor agonists, initially developed for type 2 diabetes, have shown promise in managing GDM by improving glycemic control, enhancing insulin sensitivity, and assisting in weight management. However, safety and efficacy data in pregnancy remain limited.

**Methods:**

A systematic review analyzed 8 clinical trials from ClinicalTrials.gov examining the use of GLP-1 liraglutide, semaglutide, and exenatide in GDM treatment. Studies varied in design, with the majority employing randomized, interventional protocols focusing on glycemic control and insulin sensitivity. Key outcome measures included hemoglobin A1c levels, glucose tolerance, insulin secretion, and progression to type 2 diabetes postpartum.

**Results:**

GLP-1 receptor agonists effectively manage blood glucose and reduce pregnancy complications associated with GDM. However, side effects such as gastrointestinal discomfort and mild hypoglycemia were common, and all GLP-1 are categorized as pregnancy Category C by the U.S. Food and Drug Administration (FDA), with potential implications for fetal health due to transplacental passage.

**Conclusion:**

This review highlights the need for large-scale, long-term studies to establish standardized protocols and assess the safety and efficacy of GLP-1 in managing GDM, potentially expanding therapeutic options for this condition.

## 1. Introduction

Gestational diabetes mellitus (GDM) is one of the most common complications during pregnancy, affecting approximately 7% to 10% of pregnancies worldwide.^[1–3]^ GDM is characterized by glucose intolerance that first appears during pregnancy, with adverse implications for both maternal and fetal health.^[4,5]^ Women with GDM are at increased risk of developing type 2 diabetes postpartum, and offspring of mothers with GDM may face risks of obesity, metabolic disorders, and type 2 diabetes later in life.^[6–9]^ Therefore, effective management of blood glucose levels during pregnancy is crucial for both immediate and long-term health risks.

Traditional treatment options for GDM include lifestyle modification, insulin therapy, and oral hypoglycemic agents such as metformin or glyburide.^[10–12]^ Although effective to some extent, these treatments can have limitations.^[13]^ Insulin, for instance, requires multiple daily injections, which can be challenging for patients,^[14]^ and while metformin is generally well-tolerated, its safety profile remains somewhat controversial, as it crosses the placenta.^[15]^ Therefore, there is a persistent need for alternative pharmacological treatment that can safely and effectively control hyperglycemia in pregnant women with GDM.

Glucagon-like peptide-1 (GLP-1) receptor agonists represent a newer class of antidiabetic medications originally approved for type 2 diabetes management.^[16,17]^ These medications function by activating GLP-1 receptors, which enhances glucose-dependent insulin secretion, suppresses glucagon release, and delays gastric emptying, resulting in improved glycemic control without a significant risk of hypoglycemia.^[18,19]^ Some GLP-1, such as liraglutide and exenatide, have shown potential outcomes in non-pregnant populations for improving blood glucose levels and promoting weight loss, making them a potentially appealing option for GDM.^[20,21]^ However, since GLP-1 were not initially developed with pregnancy, safety and efficacy data in pregnant remain limited. The mechanism of action of GLP-1 might benefit pregnant women by providing adequate glycemic control, while minimizing insulin resistance, which is often exacerbated during pregnancy due to hormonal changes.

Recent clinical studies and trials have begun exploring the role of GLP-1 in managing GDM, specifically examining their impact on glycemic control, maternal outcomes, and fetal development.^[22–24]^ These studies vary in design and outcomes, with some indicating potential benefits and others raising concerns about possible adverse effects on pregnancy.^[22,25]^ GLP-1 receptor agonists are currently classified as Food and Drug Administration (FDA) pregnancy category C, indicating that while animal studies have shown adverse fetal effects, adequate and well-controlled studies in pregnant women are deficient, and these agents are not recommended for use during pregnancy.^[26,27]^

This systematic review aimed to analyze clinical trial data regarding the efficacy and safety of GLP-1 in the management of GDM and to assess the current evidence on the safety and efficacy of GLP-1 receptor agonists as pharmacologic options for managing GDM, based on available clinical trial data. By examining various studies, and assessed the extent to which GLP-1 can be integrated into GDM treatment protocols, considering critical parameters such as glucose control, maternal and fetal outcomes, adverse events, and overall patient acceptability. Therefore, enhancing therapeutic strategies for managing GDM and reducing its associated risks.

## 2. Methods

On October 2024, a systematic search was conducted on ClinicalTrials.gov to identify relevant studies exploring the use of GLP-1 for managing GDM. ClinicalTrials.gov was selected as it offers a comprehensive, publicly accessible registry of interventional trials. Given the limited published data on GLP-1 use in GDM, this platform provided the most relevant and up-to-date information, including ongoing studies with detailed protocols not yet available in peer-reviewed literature.

**Figure 1.**
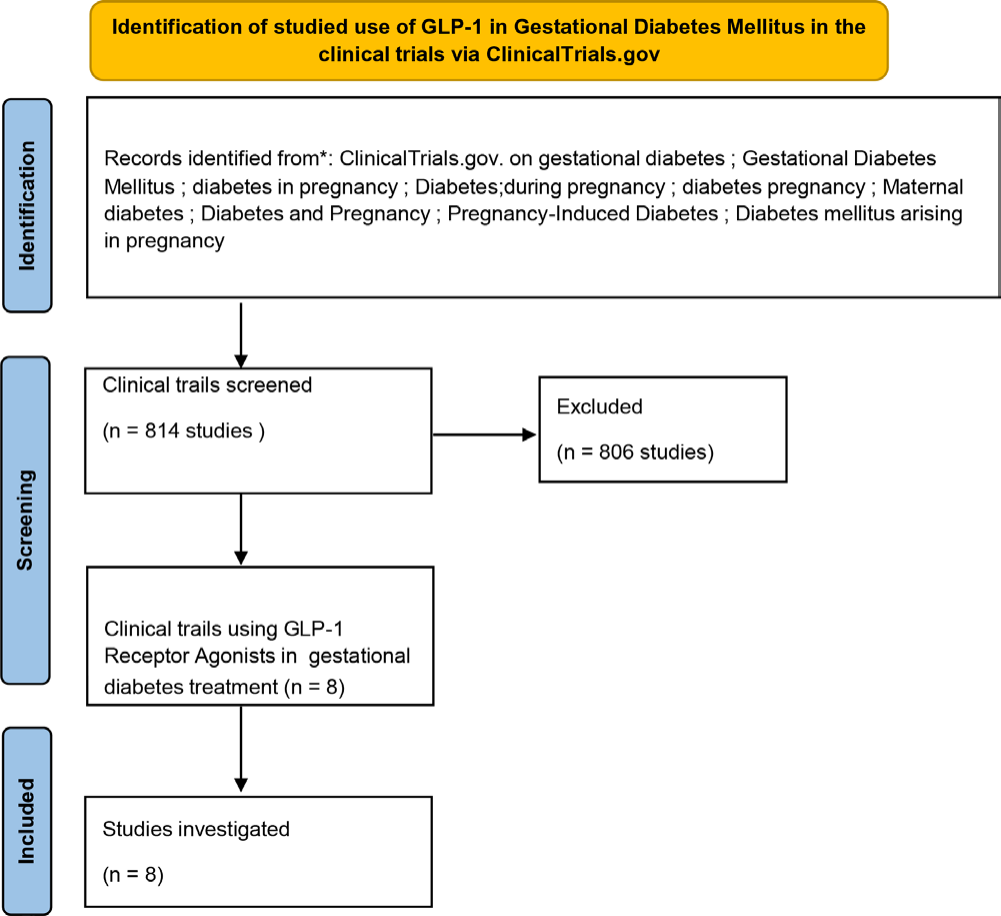
Methods used to identify studied use of glucagon-like peptide-1 in gestational diabetes mellitus.

The search used terms such as “GDM,” “diabetes in pregnancy,” “pregnancy-induced diabetes,” and similar terms associated with diabetes during pregnancy. The goal was studies focused on GLP-1 in GDM treatment. The search generated a total of 814 studies. Then, further screening process was applied, guided by inclusion criteria of all the clinical trials that were involved the use of GLP-1 as a primary intervention for GDM, where all other studies were excluded. Thus, following the screening, 806 studies were excluded, primarily due to their lack of focus on GLP-1 in GDM treatment, narrowing the selection to 8 studies as shown in Figure 1.

### 2.1. Data extraction and analysis

Data extraction focused on collecting essential study attributes to enable a detailed comparison of GLP-1 interventions in GDM characteristics as shown in Table 1. Key extracted elements included, study title, condition, intervention, primary outcome measures locations and enrolment as shown in Table 2. Each study was systematically reviewed and categorized based on its design, outcomes, and interventions. Descriptive analyses were performed to summarize the findings across trials, and trends in outcome measures were identified to assess the efficacy and safety of GLP-1 for managing GDM. Ethical approval was not required for this systematic review, as it uses publicly available, de-identified data from ClinicalTrials.gov without direct patient interaction.

**Table 1:** Characteristics of included studies on GLP-1 receptor agonists in gestational diabetes mellitus.

Characteristics	N (percentage)
Conditions	
Gestational diabetes mellitus	6 (75%)
Glucose intolerance after a recent history of gestational diabetes	1 (12.5%)
Pre-diabetes Postpartum disorder	1 (12.5%)
Phases	
Phase 1	0
Phase 2	0
Phase 3	2 (25%)
Phase 4	4 (50%)
Not available	2 (25%)
Study type	
Interventional	8 (100%)
Observational	0
Study design	
Randomized	5 (62.5%)
Non-randomized	2 (25%)
Not available	1 (12.5%)
Interventions	
Liraglutide	3 (37.5%)
Semaglutide	2 (25%)
Exenatide	2 (25%)
Other	1 (12.55)
Location	
Europe	4 (50%)
** **USA	4 (50%)

GLP-1 = glucagon-like peptide-1, USA = United States of America.

**Table 2:** Efficacy and outcome measures of GLP-1 receptor agonists in the management of gestational diabetes mellitus.

Study title	Conditions	Interventions	Primary outcome measures	Enrolment
The Impact of Liraglutide on Glucose Tolerance and the Risk of Type 2 Diabetes in Women with Previous Pregnancy-induced Diabetes	Gestational diabetes mellitus	DRUG: Liraglutide DRUG: Placebo	Change in glucose tolerance, Changes in glucose is measured by area under the curve for the plasma glucose excursion following a 4-h 75 g oral glucose tolerance test (OGTT), from baseline to 52 wk, 53 wk, 260 wk, and 261 wk	105
Visualizing Beta Cells in Patients with a History of Gestational Diabetes	Gestational diabetes	RADIATION: 68Ga-NODAGA-exendin-4 PET/CT	Pancreatic uptake of 68Ga-NODAGA-exendin-4, pancreatic tracer uptake by quantitative analysis of PET images, 1 year	24
Combined Liraglutide and Metformin Therapy in Women with Previous Gestational Diabetes Mellitus (GDM)	Gestational diabetes mellitus Type 2 diabetes mellitus Metabolic syndrome Impaired glucose tolerance Disorder of glucose regulation	DRUG: Metformin XR plus placebo DRUG: Metformin XR plus liraglutide	Insulin secretion-sensitivity index (IS-SI), IS-SI in liraglutide-metformin (LIRA-MET) therapy compared to metformin alone (PLacebo-MET), 84 wk of treatment	153
Semaglutide for the Treatment of Glucose Intolerance in Women with Prior Gestational Diabetes	Glucose intolerance after a recent history of gestational diabetes	DRUG: Semaglutide pen injector DRUG: Semaglutide placebo	Type 2 diabetes, development of type 2 diabetes defined by fasting glycemia, oral glucose tolerance test and/or HbA1c according to the ADA criteria, by 160 wk	252
Liraglutide in the Prevention of Type 2 Diabetes After Gestational Diabetes	Gestational diabetes	DRUG: Liraglutide 6 MG/ML [Victoza] DRUG: Placebos	Incidence of type 2 diabetes, assessed by fasting P-glucose \>7 mmol/L and/ or 2 h \>11 mmol/L in oral glucose tolerance test (OGTT) 75 g, and/or HbA1c ≥ 6.5%, from year 1–5	80
Exenatide Pharmacokinetics and Pharmacodynamics in Gestational Diabetes	Gestational diabetes	DRUG: Exenatide	Area under the plasma concentration vs time curve (AUC) of glucose, glucose concentration over the course of each study visit following mixed tolerance test, baseline and 1 wk later	13
Pilot Study of Exenatide Pharmacokinetics and Pharmacodynamics in Gestational Diabetes	Gestational diabetes	DRUG: Exenatide GENETIC: Buccal/blood sample collection	Glycemic control through insulin, glucose, c-peptide and glucagon assays, during testing days	0
Treatment to Regress to Normoglycemia in Women with a Recent History of GDM	Pre diabetes Postpartum disorder	DRUG: Semaglutide pen injector [Ozempic] DRUG: Placebo semaglutide pen injector	Regression to normoglycemia, glucose tolerance to be determined by glycemic response to a 75 g, 2-h oral glucose tolerance test (OGTT). Regression to normoglycemia is defined by fasting glucose \<100 mg/dL and 120 min glucose \<140 mg/dL, After 24 wk of full-dose treatment	102

ADA = American Diabetes Association, AUC = area under the curve, GDM = gestational diabetes mellitus, HbA1c = hemoglobin A1c, IS-SI = insulin secretion-sensitivity index, OGTT = oral glucose tolerance test, PET/CT = positron emission tomography/ computed tomography, P-glucose = plasma glucose, XR = extended release.

## 3. Results

### 3.1. Characteristics of included studies

A total of 8 studies were included in this review, each focusing on the use of GLP-1 in treating conditions associated with GDM and related glucose intolerance. The primary characteristics of these studies are summarized in Table 1. The majority of the studies (6 out of 8, 75%) specifically addressed GDM. Among the 8 trials, 2 studies (25%) were conducted in Phase 3, focusing on evaluating the efficacy and monitoring adverse effects of GLP-1 in a controlled environment. Four studies (50%) were Phase 4 trials, which typically emphasize safety surveillance post-market approval, indicating a focus on long-term safety and efficacy in real-world settings. The remaining 2 studies did not specify their phase, limiting interpretability regarding their progression in the clinical trial process.

All studies were interventional, meaning they actively tested GLP-1 as a treatment intervention rather than simply observing existing conditions. The majority of studies (5 out of 8, 62.5%) utilized a randomized design. Two studies (25%) employed non-randomized designs. The studies investigated a range of GLP-1 receptor agonists as shown in Table 3: Liraglutide: Used in 3 studies, making it the most frequently tested GLP-1 for GDM. Semaglutide: Investigated in 2 studies, indicating interest in newer GLP-1 for GDM. Exenatide: Also used in 2 studies, reflecting ongoing exploration of various GLP-1 options. The studies were evenly distributed between Europe and the USA, with 4 studies conducted in each region.

**Table 3:** Summary of GLP-1 receptor agonists (liraglutide, semaglutide, exenatide) in gestational diabetes mellitus.

Aspect	Liraglutide	Semaglutide	Exenatide
Mechanism of action	Enhances glucose-dependent insulin secretion, suppresses glucagon, delays gastric emptying, and promotes satiety to improve glycemic control and reduce postprandial glucose levels.	Promotes glucose-dependent insulin release, reduces glucagon secretion, delays gastric emptying, and increases satiety, supporting prolonged glycemic control and weight management.	Increases insulin secretion in response to glucose, inhibits glucagon release, slows gastric emptying, and reduces appetite, thereby aiding in postprandial glucose control.
Pharmacokinetics	Half-life: ~13 h; administered once daily via subcutaneous injection.	Half-life: ~1 wk (injectable); once-weekly injectable or daily oral formulation available.	Half-life: 2.4 h (immediate release) or ~1 wk (extended release); administered 2× daily or weekly.
Side effects	Common Nausea, vomiting, diarrhea; usually decrease over time.	Common Nausea, vomiting, diarrhea, often dose-dependent.	Common Nausea, vomiting, diarrhea; more frequent with immediate release.
	Rare Headache, dizziness, mild hypoglycemia.	Rare Headache, potential mild hypoglycemia.	Rare Dizziness, mild hypoglycemia.
Pregnancy category	Category C	Category C	Category C

GLP-1 = glucagon-like peptide-1.

### 3.2. Efficacy and outcome measures

The 8 studies assessed the efficacy of GLP-1 in managing GDM, focusing on glycemic control, insulin sensitivity, and type 2 diabetes prevention. Six studies emphasized glycemic control, measuring hemoglobin A1c and glucose tolerance, crucial for reducing pregnancy complications such as fetal macrosomia and neonatal hypoglycemia. Three studies examined insulin sensitivity and secretion, and 2 tracked type 2 diabetes progression post-GDM. Sample sizes ranged from 24 to 252 participants, with larger studies often exploring long-term preventive effects of GLP-1. Safety monitoring revealed mild gastrointestinal side effects like nausea, but no severe adverse events or negative fetal outcomes were attributed to GLP-1 exposure.

## 4. Discussion

GDM is a condition marked by glucose intolerance that develops during pregnancy and can significantly affect maternal and fetal health.^[28]^ GDM increases the risk of complications like fetal macrosomia, preeclampsia, and type 2 diabetes postpartum.^[29–31]^ Traditional treatment approaches, including lifestyle interventions,^[32]^ insulin,^[33]^ and oral hypoglycemic agents.^[34]^ Recently, a new emerging interest in GLP-1 as therapeutic options for DM from their glucose-dependent mechanism of action, which enhances insulin secretion, reduces glucagon release, delays gastric emptying, and promotes satiety, ultimately helping control blood glucose without severe hypoglycemia.^[35,36]^ Furthermore, GLP-1 can assist in weight management, a valuable attribute for GDM patients as obesity exacerbates insulin resistance and glucose intolerance.^[37,38]^ Studies on agents such as liraglutide, semaglutide, and exenatide have shown potential for effective glycemic management in GDM, but their application is limited by concerns over fetal safety and the classification of these drugs as pregnancy category C.^[23,27]^

The pharmacokinetics of GLP-1 influence dosing regimens and suitability for GDM treatment. Therefore, liraglutide is administered as a once-daily subcutaneous injection, has a half-life of approximately 13 hours, which allows for steady blood concentration throughout the day and supports effective daily glycemic control.^[39,40]^ Semaglutide is weekly injectable and a daily oral form, has a particularly long half-life of about one week in its injectable form, permitting less frequent dosing.^[41]^ The oral version provides additional dosing flexibility,^[42]^ although its application in pregnancy has been limited.^[27]^ Exenatide is administered either twice daily or once weekly, with half-lives of around 2.4 hours for the immediate-release version and approximately one week for the extended-release form.^[43,44]^ However, the immediate-release formulation’s frequent dosing may be less convenient, affecting patient compliance. This variation in pharmacokinetics across GLP-1 is critical when considering each drug’s suitability for pregnancy, as it directly impacts blood glucose control, convenience, and potential adherence in GDM management.

On the other, the side effect profile of GLP-1 is relatively consistent across liraglutide, semaglutide, and exenatide.^[45]^ Gastrointestinal symptoms, including nausea, vomiting, and diarrhea, are the most commonly reported adverse effects.^[46]^ These side effects are generally dose-dependent and reduced over time. However, due to the nausea often associated with these drugs, initiating treatment at a lower dose and gradually increasing it can improve tolerability.^[47]^ Additional, side effects include headache, dizziness, and potential for mild hypoglycemia, particularly when combined with other glucose-lowering medications.^[48,49]^ GLP-1 are generally categorized as pregnancy category C by the FDA, indicating that animal studies have shown adverse effects on the fetus, but there are no well-controlled studies in pregnant women. liraglutide, semaglutide, and exenatide cross the placenta, which raises concerns about potential impacts on fetal development.^[26,50]^ Although these drugs might show promising results in managing GDM, they are not yet approved for use during pregnancy.

Recent findings on GLP-1 have raised concerns regarding their impact on fetal development, especially considering limited human studies and substantial reliance on animal models.^[51–53]^ According to Muller et al (2023), animal studies reveal that GLP-1 like exenatide and liraglutide are associated with reduced fetal growth, delayed skeletal ossification, and skeletal anomalies, primarily in species such as rats and rabbits.^[54]^ These effects are hypothesized to result from reduced maternal food intake and weight, which often accompany GLP-1 usage. Interestingly, studies examining placental passage of exenatide and liraglutide in human cases suggest minimal to no fetal exposure shortly after administration, indicating that maternal metabolic effects, rather than direct fetal exposure, may contribute to adverse outcomes.^[55,56]^ However, in inflammatory conditions, increased passage rates were observed in animal studies, suggesting that maternal comorbidities could potentially alter fetal exposure.^[57]^ While the transplacental transfer of GLP-1 appears limited, potential indirect impacts on fetal growth and development cannot be excluded.

A key limitation of this review is the absence of formal quality appraisal using established tools such as the Cochrane Risk of Bias Tool or GRADE. This is due to the nature of the included studies, which were primarily registry-based and ongoing, with limited or unavailable outcome data. As a result, a full risk-of-bias assessment could not be performed. Future reviews incorporating completed and published trials could allow for comprehensive quality evaluation and critical appraisal. Additionally, due to the heterogeneity and incomplete outcome data of the included trials, a meta-analysis could not be performed. A quantitative synthesis will be applicable if more studies are completed and results are fully published.

## 5. Conclusion

The use of GLP-1 medications may be effective in managing GDM by improving glycemic control and insulin sensitivity. However, concerns about fetal safety and limited human data require caution. Current research underscores the need for larger, long-term studies to establish safe use in pregnancy, focusing on pharmacokinetics and fetal outcomes. If proven safe, GLP-1 receptor agonists could provide an effective alternative for GDM management, enhancing both maternal and fetal health outcomes.

## Author contributions

**Conceptualization:** Fahad S. Alshehri.

**Data curation:** Fahad S. Alshehri.

**Formal analysis:** Fahad S. Alshehri.

**Funding acquisition:** Fahad S. Alshehri.

**Investigation:** Fahad S. Alshehri.

**Methodology:** Fahad S. Alshehri.

**Project administration:** Fahad S. Alshehri.

**Resources:** Fahad S. Alshehri.

**Software:** Fahad S. Alshehri.

**Supervision:** Fahad S. Alshehri.

**Validation:** Fahad S. Alshehri.

**Visualization:** Fahad S. Alshehri.

**Writing – original draft:** Fahad S. Alshehri.

**Writing – review & editing:** Fahad S. Alshehri.

## References

[1] Buchanan TA Xiang AH. Gestational diabetes mellitus. J Clin Invest. 2005;115:485–91. 15765129 10.1172/JCI24531 PMC1052018

[2] McIntyre HD Catalano P Zhang C Desoye G Mathiesen ER Damm P. Gestational diabetes mellitus. Nat Rev Dis Primers. 2019;5:47. 31296866 10.1038/s41572-019-0098-8

[3] Dluski DF Ruszała M Rudziński G Pożarowska K Brzuszkiewicz K Leszczyńska-Gorzelak B. Evolution of gestational diabetes mellitus across continents in 21st century. Int J Environ Res Public Health. 2022;19:15804. 36497880 10.3390/ijerph192315804 PMC9738915

[4] Jovanovic L Pettitt DJ. Gestational diabetes mellitus. JAMA. 2001;286:2516–8. 11722247 10.1001/jama.286.20.2516

[5] Alfadhli EM. Gestational diabetes mellitus. Saudi Med J. 2015;36:399–406. 25828275 10.15537/smj.2015.4.10307 PMC4404472

[6] Kawasaki M Arata N Sakamoto N. Risk factors during the early postpartum period for type 2 diabetes mellitus in women with gestational diabetes. Endocr J. 2020;67:427–37. 31969529 10.1507/endocrj.EJ19-0367

[7] Bengtson AM Ramos SZ Savitz DA Werner EF. Risk factors for progression from gestational diabetes to postpartum type 2 diabetes: a review. Clin Obstet Gynecol. 2021;64:234–43. 33306495 10.1097/GRF.0000000000000585 PMC7855576

[8] Damm P Houshmand-Oeregaard A Kelstrup L Lauenborg J Mathiesen ER Clausen TD. Gestational diabetes mellitus and long-term consequences for mother and offspring: a view from Denmark. Diabetologia. 2016;59:1396–9. 27174368 10.1007/s00125-016-3985-5

[9] Wicklow B Retnakaran R. Gestational diabetes mellitus and its implications across the life span. Diabetes Metab J. 2023;47:333–44. 36750271 10.4093/dmj.2022.0348 PMC10244196

[10] Rasmussen L Poulsen CW Kampmann U Smedegaard SB Ovesen PG Fuglsang J. Diet and healthy lifestyle in the management of gestational diabetes mellitus. Nutrients. 2020;12:3050. 33036170 10.3390/nu12103050 PMC7599681

[11] Wong VW Jalaludin B. Gestational diabetes mellitus: who requires insulin therapy? Aust N Z J Obstet Gynaecol. 2011;51:432–6. 21806589 10.1111/j.1479-828X.2011.01329.x

[12] Ryu RJ Hays KE Hebert MF. Gestational diabetes mellitus management with oral hypoglycemic agents. In: Seminars in Perinatology. WB Saunders; 2014;38:508–15. 25315294 10.1053/j.semperi.2014.08.012 PMC4252887

[13] Mpondo BC Ernest A Dee HE. Gestational diabetes mellitus: challenges in diagnosis and management. J Diabetes Metab Disord. 2015;14:42. 25977899 10.1186/s40200-015-0169-7 PMC4430906

[14] Figueroa Gray M Hsu C Kiel L Dublin S. “It’s a very big burden on me”: women’s experiences using insulin for gestational diabetes. Matern Child Health J. 2017;21:1678–85. 28092062 10.1007/s10995-017-2261-8

[15] Singh AK Singh R. Metformin in gestational diabetes: an emerging contender. Indian J Endocrinol Metab. 2015;19:236–44. 25729685 10.4103/2230-8210.149317 PMC4319263

[16] Meier JJ. GLP-1 receptor agonists for individualized treatment of type 2 diabetes mellitus. Nat Rev Endocrinol. 2012;8:728–42. 22945360 10.1038/nrendo.2012.140

[17] Nauck MA. Glucagon-like peptide 1 (GLP-1) in the treatment of diabetes. Horm Metab Res. 2004;36:852–8. 15655719 10.1055/s-2004-826175

[18] Holst JJ. Pharmacology of GLP-1-based therapies. Br J Diabetes Vasc Dis. 2008;8(2_suppl):S10–8.

[19] Spellman CW. Pharmacology of GLP-1 agonists: describing the therapeutic potential to patients. J Am Osteopath Assoc. 2011;111(2 Suppl 1):eS10–4. 21389294

[20] Buse JB Nauck M Forst T. Exenatide once weekly versus liraglutide once daily in patients with type 2 diabetes (DURATION-6): a randomised, open-label study. Lancet. 2013;381:117–24. 23141817 10.1016/S0140-6736(12)61267-7

[21] Buse JB Rosenstock J Sesti G; LEAD-6 Study Group. Liraglutide once a day versus exenatide twice a day for type 2 diabetes: a 26-week randomised, parallel-group, multinational, open-label trial (LEAD-6). Lancet. 2009;374:39–47. 19515413 10.1016/S0140-6736(09)60659-0

[22] Bonde L Vilsbøll T Nielsen T. Reduced postprandial GLP-1 responses in women with gestational diabetes mellitus. Diabetes Obes Metab. 2013;15:713–20. 23406269 10.1111/dom.12082

[23] Mosavat M Omar SZ Jamalpour S Tan PC. Serum glucose-dependent insulinotropic polypeptide (GIP) and glucagon-like peptide-1 (GLP-1) in association with the risk of gestational diabetes: a prospective case-control study. J Diabetes Res. 2020;2020:9072492. 32090124 10.1155/2020/9072492 PMC7008251

[24] Qiao L Lu C Zang T Dzyuba B Shao J. Maternal GLP-1 receptor activation inhibits fetal growth. Am J Physiol Endocrinol Metab. 2024;326:E268–76. 38197791 10.1152/ajpendo.00361.2023 PMC11193516

[25] Dumolt JH Rosario FJ Kramer AC Horwitz S Powell TL Jansson T. Maternal glucagon-like peptide-1 is positively associated with fetal growth in pregnancies complicated with obesity. Clin Sci (Lond). 2023;137:663–78. 37014924 10.1042/CS20220890

[26] Cesta CE Rotem R Bateman BT. Safety of GLP-1 receptor agonists and other second-line antidiabetics in early pregnancy. JAMA Intern Med. 2024;184:144–52. 38079178 10.1001/jamainternmed.2023.6663 PMC10714281

[27] Dao K Shechtman S Weber-Schoendorfer C. Use of GLP1 receptor agonists in early pregnancy and reproductive safety: a multicentre, observational, prospective cohort study based on the databases of six teratology information services. BMJ Open. 2024;14:e083550. 10.1136/bmjopen-2023-083550 PMC11043712 38663923

[28] Sweeting A Hannah W Backman H. Epidemiology and management of gestational diabetes. Lancet. 2024;404:175–92. 38909620 10.1016/S0140-6736(24)00825-0

[29] Panunzi C Cardinali F Khalil A. Ultrasound prediction of fetal macrosomia in pregnancies complicated by diabetes mellitus: a systematic review and meta-analysis. J Perinat Med. 2024;52:623–32. 38860644 10.1515/jpm-2024-0121

[30] Aziz F Khan MF Moiz A. Gestational diabetes mellitus, hypertension, and dyslipidemia as the risk factors of preeclampsia. Sci Rep. 2024;14:6182. 38486097 10.1038/s41598-024-56790-z PMC10940289

[31] Wang Y Wei W Guo H. Postpartum life interventions to prevent type 2 diabetes in women with gestational diabetes: a systematic review and meta-analysis. J Diabetes Investig. 2024;15:1115–28. 10.1111/jdi.14220 PMC11292388 38727771

[32] Takele WW Vesco KK Josefson J; ADA/EASD PMDI. Effective interventions in preventing gestational diabetes mellitus: a systematic review and meta-analysis. Commun Med (Lond). 2024;4:75. 38643248 10.1038/s43856-024-00491-1 PMC11032369

[33] Brown J Grzeskowiak L Williamson K Downie MR Crowther CA. Insulin for the treatment of women with gestational diabetes. Cochrane Database Syst Rev. 2017;11:CD012037. 29103210 10.1002/14651858.CD012037.pub2 PMC6486160

[34] Langer O. Gestational diabetes and oral hypoglycemic agents: a fresh look at the safety profile. OBG Manage. 2003;15:62–75.

[35] Drucker DJ. The GLP-1 journey: from discovery science to therapeutic impact. J Clin Invest. 2024;134:e175634. 38226625 10.1172/JCI175634 PMC10786682

[36] Olanrewaju OA Sheeba F Kumar A. Novel therapies in diabetes: a comprehensive narrative review of GLP-1 receptor agonists, SGLT2 inhibitors, and beyond. Cureus. 2023;15:e51151. 38283440 10.7759/cureus.51151 PMC10811430

[37] Moore PW Malone K VanValkenburg D. GLP-1 agonists for weight loss: pharmacology and clinical implications. Adv Ther. 2023;40:723–42. 36566341 10.1007/s12325-022-02394-w

[38] Myerson M Paparodis RD. Pharmacotherapy of weight-loss and obesity with a focus on GLP 1-receptor agonists. J Clin Pharmacol. 2024;64:1204–21. 38924121 10.1002/jcph.2487

[39] Alruwaili H Dehestani B Le Roux CW. Clinical impact of liraglutide as a treatment of obesity. Clin Pharmacol. 2021;13:53–60. 33732030 10.2147/CPAA.S276085 PMC7958997

[40] Kyriakidou A Kyriazou AV Koufakis T. Clinical and genetic predictors of glycemic control and weight loss response to liraglutide in patients with type 2 diabetes. J Pers Med. 2022;12:424. 35330424 10.3390/jpm12030424 PMC8955617

[41] Meier JJ. Efficacy of semaglutide in a subcutaneous and an oral formulation. Front Endocrinol (Lausanne). 2021;12:645617. 34248838 10.3389/fendo.2021.645617 PMC8269445

[42] Anderson SL Beutel TR Trujillo JM. Oral semaglutide in type 2 diabetes. J Diabetes Complications. 2020;34:107520. 31952996 10.1016/j.jdiacomp.2019.107520

[43] Johansen NJ Dejgaard TF Lund A. Effects of short-acting exenatide added three times daily to insulin therapy on bone metabolism in type 1 diabetes. Diabetes Obes Metab. 2022;24:221–7. 34617375 10.1111/dom.14568

[44] Cui H Zhao C-Y Lv Y. Safety, pharmacokinetics and pharmacodynamics of multiple escalating doses of PEGylated exenatide (PB-119) in healthy volunteers. Eur J Drug Metab Pharmacokinet. 2021;46:265–75. 33576936 10.1007/s13318-020-00665-x

[45] Consoli A Formoso G. Potential side effects to GLP-1 agonists: understanding their safety and tolerability. Expert Opin Drug Saf. 2015;14:207–18. 25496749 10.1517/14740338.2015.987122

[46] Bettge K Kahle M Abd El Aziz MS Meier JJ Nauck MA. Occurrence of nausea, vomiting and diarrhoea reported as adverse events in clinical trials studying glucagon-like peptide-1 receptor agonists: a systematic analysis of published clinical trials. Diabetes Obes Metab. 2017;19:336–47. 27860132 10.1111/dom.12824

[47] Filippatos TD Panagiotopoulou TV Elisaf MS. Adverse effects of GLP-1 receptor agonists. Rev Diabet Stud. 2014;11:202–30. 26177483 10.1900/RDS.2014.11.202 PMC5397288

[48] DeFronzo RA. Combination therapy with GLP-1 receptor agonist and SGLT2 inhibitor. Diabetes Obes Metab. 2017;19:1353–62. 28432726 10.1111/dom.12982 PMC5643008

[49] Zinman B Gerich J Buse JB; LEAD-4 Study Investigators. Efficacy and safety of the human glucagon-like peptide-1 analog liraglutide in combination with metformin and thiazolidinedione in patients with type 2 diabetes (LEAD-4 Met+TZD). Diabetes Care. 2009;32:1224–30. 19289857 10.2337/dc08-2124 PMC2699702

[50] Dogan SE Kuşkonmaz SM Koc G Aypar E Çulha C. Case series: exposure to glucagon-like peptide-1 receptor agonist in the first trimester of pregnancy in two siblings. Endocr Metab Immune Disord Drug Targets. 2024;24:1237–9. 37937565 10.2174/0118715303252109231023115112

[51] Graham DL Madkour HS Noble BL Schatschneider C Stanwood GD. Long-term functional alterations following prenatal GLP-1R activation. Neurotoxicol Teratol. 2021;87:106984. 33864929 10.1016/j.ntt.2021.106984 PMC8555578

[52] Pereira de Arruda EH Vieira da Silva GL da Rosa-Santos CA. Protein restriction during pregnancy impairs intra-islet GLP-1 and the expansion of beta-cell mass. Mol Cell Endocrinol. 2020;518:110977. 32791189 10.1016/j.mce.2020.110977

[53] Ma XL Ding Y Wu L-M. The glucagon-like peptide-1 (GLP-1) analog exenatide ameliorates intrauterine adhesions in mice. Peptides. 2021;137:170481. 33450323 10.1016/j.peptides.2020.170481

[54] Muller DRP Stenvers DJ Malekzadeh A Holleman F Painter RC Siegelaar SE. Effects of GLP-1 agonists and SGLT2 inhibitors during pregnancy and lactation on offspring outcomes: a systematic review of the evidence. Front Endocrinol (Lausanne). 2023;14:1215356. 37881498 10.3389/fendo.2023.1215356 PMC10597691

[55] Hiles RA Bawdon RE Petrella EM. Ex vivo human placental transfer of the peptides pramlintide and exenatide (synthetic exendin-4). Hum Exp Toxicol. 2003;22:623–8. 14992323 10.1191/0960327103ht402oa

[56] Dao K Shechtman S, Weber-Schoendorfer C, . Use of GLP1 receptor agonists in early pregnancy and reproductive safety: a multicentre, observational, prospective cohort study based on the databases of six Teratology Information Services. BMJ Open. 2024;14:e083550. 10.1136/bmjopen-2023-083550 PMC11043712 38663923

[57] You L Deng Y Li D Lin Y Wang Y. GLP-1 rescued gestational diabetes mellitus-induced suppression of fetal thalamus development. J Biochem Mol Toxicol. 2023;37:e23258. 36424357 10.1002/jbt.23258

